# Critical Self-reflection on Racism by Hospital Physicians in Large German Cities. A Qualitative Reconstructive Study Using Episodic Interviews

**DOI:** 10.1007/s40615-024-02073-2

**Published:** 2024-07-05

**Authors:** Tonia Nassal, Hürrem Tezcan-Güntekin

**Affiliations:** 1 Department Public Health, Berlin School of Public Health: Charité - Universitätsmedizin Berlin, Alice Salomon Hochschule, Technische Universität Berlin, Charitéplatz 1, 10117 Berlin, Germany; 2 Department of Public Health, Berlin School of Public Health: Charité - Universitätsmedizin Berlin, Alice Salomon Hochschule, Technische Universität Berlin, Charitéplatz 1, 10117 Berlin, Germany; 3 Department Public Health, Alice Salomon Hochschule Berlin, Alice-Salomon-Platz 5, 12627 Berlin, Germany

**Keywords:** Racism, Critical whiteness, Self-reflection, Physicians

## Abstract

Racism permeates healthcare institutions and interpersonal interactions, impacting both staff and patients. The role of doctors, given their influential position in the healthcare system, is particularly crucial in this context. Despite this, there is a scarcity of evidence regarding the manifestation of racism among healthcare professionals in Germany. Critical whiteness studies emphasize the importance of white* individuals engaging in critical self-reflection to mitigate racism. This study aimed to explore the attitudes of white* physicians in hospitals in major German cities towards racism and their critical reflection on personal attitudes and actions concerning racism in interactions with staff members and patients. Data was collected through six episodic interviews with physicians, analyzed using the reconstructive qualitative procedure of the documentary method, leading to a sense-genetic typology. The sense-genetic typology revealed three distinct attitudes towards racism: acknowledging, individualistic, and ignoring. Four types emerged concerning the self-reflection of white doctors: self-critical, socially critical, worried, and defensive. The most promising potential for interventions to reduce racism lies within the self-critical and socially critical types, both demonstrating an acknowledging attitude. Conversely, the worrying and defensive types may present challenges in deconstruction. This suggests that interventions aimed at reducing racism should be tailored and implemented with a nuanced approach.

## Introduction

International studies consistently highlight the detrimental physical and mental health effects of racism [1–4]. However, despite this evidence, racism persists within the healthcare system at systemic and interpersonal levels [5], affecting both patients [6, 7] and staff [8–10]. An illustrative example is the unequal pain treatment for Black patients in the US, rooted in the bias that Black individuals have a higher pain tolerance [11]. Similar stories emerge in Germany, where patients face unequal treatment due to the stereotype of “Morbus Mediterraneus,” implying that individuals associated with the Mediterranean region are more theatrical in reporting pain [12].

Research on racism in healthcare is predominantly conducted in the US, followed by Canada, Australia, and the UK. Evidence from other European countries is still widely lacking, particularly concerning racialized individuals who are not refugees or recent immigrants (native-born or non-immigrants) [13]. This paucity is attributed to the post-war rejection of using the category “race” as a scientific category [14] and the denial of the ongoing existence of racism in Germany [15]. Even though the high sensitivity and taboo in Germany surrounding discussions on racism have proven ineffective in addressing the underlying issue of racism’s existence [16, 17], the majority of studies persist in directing their focus on the experiences of individuals with a migrant background rather than on racism itself [18]. Although these two concepts are often associated, they cannot be equated [19]. However, existing evidence about disparities in healthcare access and utilization based on social group or migration status indicates that racism in the healthcare sector in Germany and other EU countries is comparable to the international stage [18, 20]. Recent efforts in this country to focus on racism are underway to bridge this knowledge gap [13, 21]. While some initiatives continue to rely on migration-based surveys for certain aspects [21], others aim to address this lack of focus on racism and are striving to provide deeper insights into the experiences, situations, and interpretations of racism and racial discrimination in healthcare institutions in Germany [13]. The necessity of focusing on the racist attitudes of healthcare staff in Germany has yet to be addressed [18].

Drawing on critical whiteness studies, this research places the responsibility for deconstructing racism on white*^1^ individuals who benefit from it, often exhibiting subtle and unconscious forms of racism and perpetuating structural racism [16]. This study delves into the attitudes and self-reflection on racism among white* physicians in hospitals in major German cities. This focus aims to unveil distinct implications for deconstructing racism among physicians, providing essential knowledge for designing targeted interventions to reduce racism and enhance the well-being and opportunities for racialized patients and healthcare professionals [22].

## Background

Racism is recognized as a critical social determinant of health due to its documented adverse effects on individuals’ physical and mental well-being [23–26]. Given its profound impact, racism should be acknowledged as a significant public health concern [23]. In healthcare settings, the presence of racism exacerbates health disparities [27]. International studies emphasize that systematic and interpersonal racism towards patients significantly influences healthcare utilization, trust, contentment, and perceived quality of care [28, 29]. Patients reporting perceived racism often describe experiences of microaggressions, encompassing everyday humiliations through verbal statements, actions, or environmental factors [30]. These experiences manifest as longer waiting times, shorter doctor-patient interactions [31], speech barriers [18], and feelings of not being taken seriously or respected [32, 33]. Implicit racist attitudes and actions of physicians further impact patient-doctor communication, potentially leading to unequal diagnosis and treatment [11, 34–36].

Systematic and interpersonal racism directed at health workers negatively affects their careers and health [10, 37]. Staff members report encountering microaggressions from colleagues and patients, along with a denial of their professional competence due to racist prejudices [10, 38, 39]. Additionally, affected staff often feel a lack of recognition of racism by colleagues and healthcare institutions, leading to a sense of not being heard, supported, or respected [10].

In Germany, the influence of structural racism is evident in the demographic composition of doctors, most of whom are still white* [16]. Their predominantly white* status, coupled with their high income [40] , places them in a particularly privileged position in society. Physicians bear significant responsibilities in their decisions towards patients, wielding considerable power over both patients and staff [41]. Consequently, their attitudes and actions carry substantial consequences. The challenging work environment in hospitals, characterized by time pressure, frequent interruptions, stress, and exhaustion, poses a risk of shaping individuals’ actions based on prejudices [31, 42–44]. Given these conditions are prevalent in hospitals [43, 45], physicians working in these settings emerge as crucial target groups for initiatives aimed at reducing racism in the healthcare system. As 58.6% of people with a migration background in Germany resided in cities in 2022 compared to 13.4% in rural areas [46], physicians working in large cities are likely to encounter racially marked individuals as both colleagues and patients. Consequently, their attitudes and actions towards racially marked people become particularly relevant for comprehensive interventions. While international studies predominantly adopt quantitative approaches to investigate racial prejudices and biases among physicians [6, 31, 47, 48], there is a noticeable gap in the qualitative exploration of critical self-reflection on racism among physicians, a crucial aspect for effective racism reduction [22, 23].

## Research Aims

The primary objective of this research project was to contribute to the understanding of various attitudes towards racism and degrees of self-reflection among white* physicians working in hospitals in major German cities. Despite being identified as a research gap by the “Antidiskriminierungsstelle des Bundes,” information on the racist attitudes of healthcare professionals, to the authors’ knowledge, has not been collected in this country.

Aligned with the critical whiteness studies, this study places the white*, unnamed individual—typically in the role of researching and describing others—as the object of investigation. The study sought to ascertain the extent to which white* physicians are cognizant of the racism experienced by a significant proportion of patients and staff in the hospital. The rationale behind this choice lies in the belief that, to effectively reduce racism, it is crucial for individuals involved in its (re-)production to possess awareness of racism in general, and more specifically, their own contributions to it. It is assumed that this awareness is a pivotal step towards deconstructing ingrained patterns of thinking, speaking, and acting, often entrenched in unconscious biases. The overarching purpose of this study is to establish an evidence base that informs the understanding of different attitudes and reflexive practices of physicians concerning racism. This insight is intended to provide a foundation for gauging the potential for deconstructing racism and underscore the need for nuanced interventions to reduce racism within hospital settings.

## Methods

The determination of prioritized topics of this study was informed by a scoping review examining the current state of research and identifying gaps. Additionally, insights from authors within critical whiteness studies were considered in defining the research focus. The present research work is based on the reconstructive qualitative research design of the documentary method according to Bohnsack [49]. Due to the highly elaborate analytical method, the number of six interviews was considered a realistic goal for this study.

Ethical approval for the study was obtained from the Charité—Universitätsmedizin Berlin’s ethical committee on June 20, 2022. The recruitment of participants involved reaching out to various hospitals in large German cities, the medical journal “Ärzteblatt” from every German state, and individuals acquainted with the authors working in the hospital. Those contacted were requested to share the Call for Participation in the study. Only physicians unknown to the authors were considered for participation. Inclusion criteria mandated that participating physicians self-identify as white*, actively work in a hospital in a large German city (with a population of at least 100,000 inhabitants), and are proficient in the German language. Prior to conducting interviews with physicians, comprehensive information about the research project was provided and written consent was obtained from each participant. The first six physicians who met the inclusion criteria and consented to participation were interviewed. The interviews were conducted using Flick’s method of “episodic interviews” [50]. This approach involves posing semantic questions aimed at eliciting a person’s semantic knowledge, followed by one or more episodic questions intended to elucidate the individual’s experience-based knowledge [50]. This methodology is therefore well-suited for reconstructive analytical approaches and particularly effective for the documentary method’s goal of explicating the implicit experiential realm of an individual. Recognizing that a person’s life experiences are best captured through free narrative, the interview structure was designed to be flexible, allowing for various follow-up questions. This semi-structured interview guide comprised 16 questions, divided into different sections: the first part focused on racism, the second on privilege, and the third on power (the interview guide can be found in the Appendix).

The one-to-one interviews were conducted via GDPR^2^-compliant video platforms. These interviews took place between July 2022 and August 2022, with each session lasting approximately 30 to 40 min. All interviews were recorded for transcription and analysis. The transcription and subsequent analysis followed the documentary method outlined by Bohnsack [49]. Transcription was done by hand by one of the authors using the MAXQDA-Software. Both authors were involved into the reconstructive analysis which comprised the following three steps. In the first step, all statements were formally summarized without interpretation, and main themes and subthemes were organized. Essential passages relevant to addressing the research questions were identified for further analysis [51]. The second step, termed “reflective interpretation,” involved semantically and comparatively analyzing the marked passages, considering how something is said and comparing statements on the same topic within the same interview and across different interviews. The analysis was conducted with respect to the interviewee’s frame of orientation, avoiding judgment about the veracity of statements and instead focusing on what the statements reveal about the interviewee’s attitude or view [51]. The final step involved creating “sense-genetic types” of attitudes and self-reflection which are detached from specific cases; the abstraction from individual cases refers to the formation of each type through the aggregation of statements from various interviews or individual cases. This also implies that multiple types can be represented by various statements from a single individual [52]. Abstracting types from individuals becomes easier with a larger number of cases; with fewer interviews, more types tend to be unique to a single person and thus are not fully detached from them [52].

The types of “attitudes” refer to the various perspectives individuals can have on racism, specifically regarding the extent to which and the manner in which they perceive it as a problem. In contrast, “self-reflection” pertains to the extent to which individuals recognize and understand their own roles in the issue. The analyses of certain questions have thus led to the development of attitude typologies, while others have contributed to the formation of self-reflection typologies.

### Roles of Researchers

Tonia Nassal is a practicing physiotherapist who routinely observes everyday racism in healthcare settings. Holding an MSc in Public Health, she has engaged in scientific research focused on racism within the healthcare system. As a white* researcher, she is in an ongoing process of addressing her own racism. It is important to acknowledge that her whiteness* likely influenced the openness of the interviewees, as white* individuals tend to feel more comfortable discussing racism with other white* individuals [17]. Hürrem Tezcan-Güntekin is a sociologist and a professor with expertise in qualitative methods in public health. Her research focuses on diversity and health, transnational health practices, and racism in healthcare. In her Master’s degree courses in Public Health at Charité Berlin, she consistently addresses the impact of various forms of discrimination on health. Although she has a migration background and personal experiences with racism, she is frequently perceived as white and thus experiences “passing,” meaning that many barriers faced by other BiPoC do not apply to her. Both authors recognize that their extensive study of racism has shaped their viewpoints, which may differ from those of the study participants. The documentary method according to Bohnsack helped to mitigate the potential impact of their perspectives in the analysis of the interviews.

## Results

The findings of this research reveal significant variations in physicians’ attitudes towards racism, encompassing diverse perspectives on its prevalence and impact. Attitudes range from recognizing racism as a pervasive issue, present on multiple levels in daily interactions, to asserting that racism is not a substantial problem and goes unnoticed in the hospital setting. Consequently, the extent of self-reflection among physicians varies, influencing their awareness and evaluation of personal racist tendencies. This diversity in attitudes and associated self-reflection underscores distinct potentials for deconstructing racism. The subsequent presentation of results explores this spectrum of potential across the six individuals interviewed. Comprehensive details about their characteristics and medical positions can be found in Table 1. The abstraction of types facilitated the identification of three distinct categories related to attitudes towards racism and four different categories pertaining to self-reflection. To enhance readability, the provided quotes below have been refined.

**Table 1:** Characteristics of the participants

	Attendee 1	Attendee 2	Attendee 3	Attendee 4	Attendee 5	Attendee 6
Age	44	26	29	59	29	54
Gender	Female	Male	Female	Male	Female	Male
Medical position	Specialist	Assistant physician	Assistant physician	Chief physician	Assistant physician	Senior physician

### Attitude

#### Acknowledging Type

Racism is observed on an interpersonal as well as an institutional level. The mechanisms at play in society and the associated privileges are recognized and acknowledged.

[the prejudice] that people from the Mediterranean region are, in quotation marks, very sniveling. (A2, L. 72–73)^3^

The senior physician came from Sierra Leone, […] and when he arrived, they were all so surprised, right? […] That was a negative surprise, I must say. He always had to justify himself first and prove his competence before gaining respect (A6, L. 81–90)

During night duty, Filipino nurses are sometimes told by other experienced nurses [...] “you have to make sure they pay good attention to the patients because they may not fully understand us, they just say ‘yes, yes’ and smile.” (A3, L. 15–18)

Racism is perceived as a challenge within the broader society, often (re-)produced unconsciously.

Because I think that most of it is unconscious, this *‘Unconscious Bias’,* where one plainly has prejudices of which one is not really aware of, it is pretty widespread in our society. (A5, L. 122–124)

The hospital is subject to critical scrutiny in terms of racism and power dynamics: There is a recognized need to mitigate racism, and the institution’s or colleagues’ perceived lack of efforts in addressing it is noted and criticized (A2, 3, 5, 6).

The institution where I work […] is quite autocratic and somewhat pseudo-reflective and […] they fail to recognize a significant need […] to engage in deep self-reflection and work towards personal growth. (A6, L. 161–167)

A notable concern associated with the occasional absence of effort is identified in the hierarchical structures and the composition of leadership positions within the hospital, predominantly occupied by white* men. Privileges are linked to social determinants such as gender and race (A2, 3, 5, 6).

Often, I have the feeling that it’s a […] multilayered problem, which is also […] often connected to the hierarchic structures in the hospital and […] a lot of people who are in leading positions […] simply lack consciousness. These are mostly men […] - who do not have a migrant background - in any leading positions and obviously don’t see problems that maybe somehow less privileged people would see. (A2, L. 334–341)

The obligation to dismantle racism is recognized as incumbent upon white* individuals, acknowledging their role in challenging and dismantling systemic racial biases and structures. (A2, 6).

Finally yes, it would of course be optimal if people would also reflect on themselves and their actions essentially or reflecting on the structure in the hospital and trying to make changes there. (A2, L. 322–324)

#### Individualistic Type

In this perspective, racism is predominantly perceived as the actions of individual offenders. The recognition is limited to instances of interpersonal racism within the hospital (A1). Racism is regarded as reprehensible, often attributed to right-wing individuals, and is consequently linked to intent: an act is deemed racist only if racially motivated.

If there are actually comments that are racist towards the patients, then I clearly say, ‘that is so filthy right-wing radical, I can’t stand it.’ (A1, L. 153–160)

So when you say “where are you from?” and you don’t mean that at all in a racist way [...] and that is immediately somehow judged as racism. (A1, L. 141–143)

Racism is not perceived in relation to the power dynamics it generates. It appears to be conceptualized merely as an individual experience, devoid of significant societal consequences. This perspective implies that racism can also be perceived by white* individuals (A1).

I experienced racism myself for the first time when I was in Switzerland, [...]. I found that quite fascinating (A1, L. 127–128)

The accountability for unpleasant situations related to racism is attributed either to individuals who deliberately express or act in a racist manner (refer to quote above A1, L. 153–160) or to individuals who are racially marked and are considered overly sensitive in their reactions (refer to quote above A1, L. 141–143).

Sometimes you get the sense that it takes up a lot of space; when so many people practically demand your attention often asking similar questions. (A3, L. 42–44)

#### The Ignoring Type

In this perspective, no instances of interpersonal or institutional situations are acknowledged in which patients or staff encounter racism (A4).

That employees experience Racism? yes, so I don’t observe that myself (A4, L. 9).

Instances of racism are only acknowledged when formal complaints are filed. These complaints are generally dismissed as unjustified, reflecting the perspective that racism is not considered a significant issue within the hospital. There is a prevailing belief that individuals who raise complaints do so with specific motives. While the conduct of those who have been racially de-labeled is perceived as appropriate and rationally justifiable, the behavior of those filing complaints about racism is viewed as irrational, burdensome, and “ungrateful” (A4). Racialized individuals who voice concerns are held accountable for unpleasant situations related to racism (A1, 3, 4), and there is a lack of recognition of the need to deconstruct racism (A4).

The patient […] mentioned that he speaks German poorly; we then suggested getting someone to translate so he could understand the information, thereupon he quickly became very abusive, accusing the staff member of xenophobia […] subsequently, he left and did not return as he had announced […] yes, these situations are very stressful and time-consuming for us, taking away time from other patients. We sometimes have to endure insults, even though patients come to us seeking assistance, you know? It's just an extremely thankless situation (A4, L. 56–72)

Racism is not associated with privilege (A1, 4).

Privileges are very specific advantages […] some we may have earned, but others we simply enjoy because we live in Western Europe and not in another part of the world, […] we get to enjoy these benefits without necessarily having earned them, so to speak (A4, L. 133–137)

### Self-reflection

#### Self-critical Type

The self-critical type is characterized by the recognition and reflection upon one’s own racism. This individual can provide personal examples (A1, 3, 6), holds the conviction that they play an active role in the (re)production of racism, and acknowledges personal responsibility for its deconstruction. However, this process is perceived as ongoing and not yet complete. Criticism is welcomed, prompting thoughtful reflection.

I often inquire about people’s origins […] and if the response is “from [German city]” then that’s the correct answer and the question is wrong. […] it’s the kind of situation that leaves me annoyed like hell; it’s this kind of situations where I realize that I still have a long way to go. (A6, L. 137–143)

The recognition that structural circumstances within the hospital and the limitations in training content have a negative impact on one’s actions, potentially contributing to personal prejudiced behavior, is acknowledged (A3). Resolutions and expectations for practicing good medicine often cannot be fully implemented (A3).

But it is challenging to deal with it in everyday life or to have the understanding […] for it during stressful situations. (A3, L. 105–106)

We had a patient with a dark skin color, and it was challenging for me to make a clinical assessment of his leg, because we simply have no experience with how an ischemic (poorly perfused) leg - It's cold, bluish, and pale. But how does it look now with a person of color? (A3, L. 62–66)

Perceiving oneself as hierarchically subordinate, there is a recognition of the challenges associated with taking anti-racist action (A2).

What I consistently observe in myself is that because these hierarchies exist, […] I find it more challenging to take action about it. (A2, L. 343–345)

To actively dismantle racism in general and address one's own biases, there is an expressed desire for a more critical approach to and discourse on racism in educational and healthcare settings (A2, 3, 5).

[My desire is] for the subject to be scrutinized more critically in the hospital and for it to be perhaps even more prominently addressed in studies. […] and I believe that this is crucial […]. In self-reflection: how can I work on it? It requires open discourse; open communication. (A5, L. 298–306)

#### Socially Critical Type

A distinctive feature of this type is the consideration of racism in the context of its social and structural mechanisms. Reflection extends to assessing how institutional structures within the hospital and training programs may contribute to one’s prejudiced or racist behavior. Nevertheless, it poses a challenge to discern where one actively participates in the (re)production of racism. Recognized instances of personal racism are attributed to “objective” factors, such as language barriers, cultural differences, time pressure, or everyday stress (A2, 3, 5).

Especially from Arab families and Muslim families, which I already believe is a cultural difference, who, for example, always come to visit with a lot of people. […] Relatives often call; then we naturally always have a problem with language barriers […] Sometimes you don’t have time for it in everyday life, but you also notice that you are much more annoyed by the fact that you are so often disturbed in your work. (A3, L. 27–33)

The primary identification of one’s role in the (re-)production of racism lies in the reluctance to criticize those perpetuating racism when witnessing it. Consequently, the task to contribute to the deconstruction of racism involves a commitment to change this behavior. This initiative is deemed necessary due to the perception of racism as a pervasive social phenomenon often manifested in unconscious actions (A2, 5). A power-critical perspective emerges, highlighting the difficulty of speaking up to superiors about racism in the subordinate position as a resident due to fears of negative consequences (A2, 5).

As far as higher ranks are concerned, I would say […] that I have already thought about senior physicians and felt like, “okay, that’s completely absurd what you’re saying”, […] in the end I did not dare to say anything and was […] kind of annoyed, but it is […] finally easier to say nothing as a subordinate person who doesn't want to mess up with people […] I have now at least resolved that when such things occur again, to say something, even if it can have disadvantages for me personally (A2, L. 148–161)

The desires concerning racism involve fostering heightened critical reflection, promoting open dialogue, and cultivating an appreciative culture of learning, both in society and within the hospital setting (A2, 3, 5).

So in the end what I wish for is nothing different in the hospital than in society, just an awareness that racist structures ultimately exist […] and that […] many people benefit from this without realizing it (A2, L. 317–325)

That […] there is a way of dealing with each other; that people can express things to each other when they notice something, without it being dismissed or met with incomprehension, but rather that somehow people try to appreciate each other somehow and respond to it (A 2, L. 326–331)

#### Worried Type

Typical of this type is the preoccupation with the fear that others might perceive one as holding racist views. If one’s remarks or actions are deemed racist, despite lacking any intentional racial bias, the individual feels these judgments are unjustified (A1).

I’ve often faced insults like, ‘Yes, that’s just because I’m not German. That’s why I have to wait so long,’ and so on; that is simply, I must say, not justified […]. I think you have to look at it more in a differentiated way, because there is already a time delay due to the language barrier […] but it’s just that, for me it is never intentional, I don’t make anyone wait on purpose who can’t speak German. Yes, they are all the same. (A1, L. 79–93)

This perception instigates the fear of making mistakes. Upon recognizing one’s own racism, one experiences remorse and anxiety about unintentionally behaving in a racist manner. This heightened insecurity in interactions with racially marked individuals prompts a concerted effort to consistently communicate and act in a manner deemed “correct.” Consequently, changes in expressions and behaviors stem more from fear than a comprehensive understanding of racism’s modes of action and consequences. The dissonance between not comprehending why certain actions are perceived as racist and simultaneously attempting to avoid being labeled a racist generates a sense of strain (A1).

One is very afraid of being racist without wanting to be racist (A1, L. 107–108).

We try very hard to ensure that we're doing everything right, saying everything right, and pronouncing everything right, that is sometimes a bit of an effort because often you don't mean anything bad, […] so, I find that super exhausting at times. (A1, L. 136–145)

There is a desire for more considerate feedback and a more “relaxed” approach to racism (A1).

I would wish that my attention is drawn to what is perceived as racist and that this is then actually formulated not as 'this is racist' but as 'this is perceived as racist', because my intention behind this is not that this is racist, […] I then understand why, why I am perceived as racist, although I have primarily not meant this in a derogatory way. (A1, L. 325–332)

#### Defensive Type

This type is characterized by the conviction that racism has nothing to do with one’s person: One is sure that one does not act racist oneself. In response to the question, “Do you think you act or speak in a racist way at times?” one attendee answered with…

Through my person? I try to keep this uhm out of my actions and doings as far as I can control it myself (A4, L 76–77)

Complaints of racism are typically regarded as unjustified and often labeled as unacceptable hostility. The individuals raising concerns about racism are perceived as perpetrators, while oneself and other employees, to whom the complaints are directed, are seen as victims (A4).

Sometimes, there’s this tendency to say, ‘Yes, this is again an accusation of racism, um, which I cannot accept and which is always chosen in order to, so to speak, reject the blame from yourself and yes, to accuse others (2).’ [...] That fits - yes, reinforces itself for me almost every day (A4, L. 37–51)

As a victim [...] of accusations that accuse me of xenophobia [...]. I have had to deal with it again and again (A4, L. 110–113)

An exchange about racism with colleagues is generally not deemed necessary. In instances of such exchanges, there is a lack of mutual consideration for racism-related complaints; instead, individuals tend to shield themselves from external “attacks” (A4).

This usually happens in smaller circles when there are accusations against individual employees. We then reflect on this again in the form of a case discussion, but it's not discussed in a larger group. However, a personal discussion atmosphere among colleagues is preferred, also to maintain some personal protection for the accused colleagues (A4, L120–124)

Due to the conviction that one adequately addresses racism and plays no active role in it, one’s desires primarily concern the actions of others (A4).

I personally think I deal with it a lot [...] I would be very happy, if this were the case with all colleagues (A4, L. 211–213)

## Discussion

**Fig. 1:**
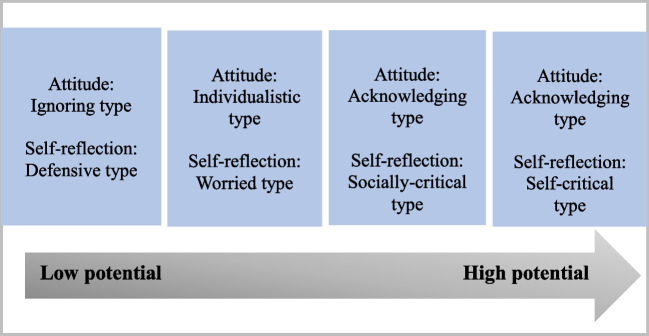
Identified types and their potential to deconstruct racism

A diverse spectrum of attitudes and levels of self-reflection on racism among physicians in German hospitals was identified, demonstrating varied potential for the deconstruction of racism. The observed attitude appeared to be correlated with one’s self-reflection (see Fig. 1).

The types identified in this study exhibit significant parallels with the attitudes and phases of self-reflection among white* individuals described in the current literature on racism critiques [17, 53]. It is important to understand these types not as distinctly separate categories, but rather as positions along a spectrum, where one attitude can closely resemble another or differ significantly.

The ignoring attitude appears to result in a defensive mode of self-reflection, with the lowest immediate potential for deconstruction of racism due to a lack of consciousness about the issue. This attitude reflects that racism is neither acknowledged on a structural nor on an interpersonal or individual level. The resulting lack of self-critical reflection which shows among others in the victim-perpetrator reversal obstructs recognizing the need for and motivation to take action. The individualistic type of attitude is connected to the worried type of self-reflection. This attitude, in which racism is linked to intention, shows a lack of understanding of racism as a social phenomenon and its modes of action. The predominant fear of being perceived as racist without meaning to be hinders self-critical questioning, making it challenging to recognize and accept one’s own subtle racism. In defensive statements of this type, unlike the defensive type, the aim is not to deny that one’s actions could be perceived as racist by others. Instead, the focus is on ensuring that one is not seen as intentionally racist by the other party. Since racism is nevertheless considered a major problem that needs to be addressed, these types of attitudes and self-reflection are associated with a greater potential for deconstruction than the ignoring and defensive type. The defensive and worried types identified in this study mirror characteristics of “white fragility” conceptualized by DiAngelo [53], indicating a result of socialization into the dominance of whiteness*. In contrast, the self-critical and socially critical types align with an acknowledging attitude, representing a shift in the racial paradigm [53]. This attitude is deemed most potent for deconstructing racism as it acknowledges the structural, interpersonal, and individual presence and modes of action of racism. Physicians embodying this attitude can identify necessary changes on various levels. The self-critical type holds the greatest potential for deconstruction, as it involves not only recognizing and acknowledging racism on both a systemic and interpersonal level (as does the socially critical type), but also entails an awareness of one’s own racism and its often subtle and unconscious manifestations. Such recognition has a high potential to change personal habits. This process is crucial for physicians to minimize the impact of unconsciously biased interactions with patients and staff members [54, 55].

In Germany, there is a lack of studies focusing on investigating physicians’ attitudes regarding racism [13, 18]. International studies in this context primarily examine whether implicit or explicit racial biases are present among physicians and how these biases influence their decision-making and patient-physician interactions, and contribute to exacerbating health inequality for BIPoC (Black, Indigenous, and People of Color) [6, 31, 35, 47, 48, 56]. These studies demonstrate that implicit racial biases are especially prevalent [6, 31, 47], although the results regarding the effects of these biases vary widely. Increasingly, studies on the international agenda are examining specific beliefs related to racism and their impact on medical practice [11, 36, 57]. The present study shows that attitudes consistent with “white fragility” align with these implicit racial biases found in international research and reveal specific beliefs connected to these biases. This is evident in racially biased behavior attributions, such as labeling racially marked individuals as “hypersensitive” or engaging in victim-blaming by attributing malicious behavior to these individuals.

Although calls for studies employing more complex methods to assess racist attitudes among physicians [6, 31] are lately being addressed, these studies predominantly use quantitative methods [11, 34–36, 56]. Qualitative methods are particularly valuable for research topics with a high degree of subjectivity, where the individual is both the starting point and the aim of the research [58]. Awareness of racism [59] and the recognition and acceptance of one’s own stereotypes and prejudices are necessary for motivation and active engagement in deconstruction efforts [54, 55].

This study, therefore, not only addresses the knowledge gap about racism in German healthcare but also contributes to the international agenda by investigating attitudes and self-reflection on racism using qualitative methods. It provides insights into the extent of awareness regarding the mechanisms of racism, the power dynamics, and white privileges it produces, and the consciousness of one’s own racism.

Hassen et al. show that effectively addressing racism in healthcare necessitates structural, interpersonal, and individual changes within universities and health institutions [22]. Prior to developing targeted interventions, it is essential to identify specific needs and considerations for implementation [22]. The findings of the present study suggest that different attitudes and levels of self-reflection influence the acceptance of interventions targeting individual, interpersonal, and structural changes. Physicians who correspond to the self-critical type are likely to welcome these interventions, including those aimed at changing their own behaviors. In contrast, those who correspond to the defensive type are likely to react with rejection, anger, and overall lack of acceptance. Such reactions have been frequently observed in previous mandatory interventions aimed at reducing racism at the individual level [60]. For individuals who demonstrate a defensive response to racism, it would likely be beneficial to first implement interventions that foster a positive attitude. Examples of such interventions could include creating positive contact with Black people and people of color [59, 61] and conducting workshops or training sessions that raise awareness of the mechanisms and consequences of racism, including white privilege and power [55, 60, 62, 63]. Only after this can the essential need for critical reflection on one’s own attitudes and beliefs regarding racism [42, 55] be addressed.

Addressing individual racism is regarded as a key strategy to mitigate institutional racism, ensuring the importance of promoting BIPoC representation in leadership roles [22, 64] is recognized and supported. Recommendations also highlight the need to improve the educational system to address BIPoC underrepresentation in the medical profession and higher hierarchical positions. This includes enhancing licensure procedures, providing financial aid, tackling interpersonal discrimination, and incorporating anti-racism content into the curriculum [37]. Additionally, structural interventions should implement laws, policies, strategies, and workflows that address racism [22], such as an easily accessible, multilingual complaint and counseling structure [13]. Merz et al. emphasize improving overall working conditions to reduce racial discrimination, given the increased reliance on prejudices under stressful conditions. Before implementing anti-racist interventions, Hassen et al. highlight the need for establishing basic conditions like an explicit, shared language of anti-racism, resources, financing, and expertise. Transparency and accountability mechanisms should be considered, and such interventions should be continuously monitored and evaluated [22].

When considering the results of this research study, the following limitations must be taken into account: Due to the nature and objective of the research, as well as the method of data collection, it is likely that only physicians who are willing to engage with issues regarding racism and dedicate their time to participate have taken part in the study. Consequently, the results of this sample probably do not reflect the heterogeneity of attitudes among all hospital-based physicians in Germany. Additionally, it should be noted that due to the extensive analysis required by the documentary method, only six interviews could be conducted for feasibility reasons. Although this abstraction method allows for generally valid statements to be made, even with a smaller sample size compared to some other qualitative methods [49], this cannot be fully attained with only six interviews. Instead, the results obtained through this study allow for a deeper examination of various attitudes towards racism, revealing different lines of argumentation, their implications, and their potential for deconstructing racism.

Because of the limitations of this study’s findings, further research with a larger sample size and an expanded scope of participants is necessary. This includes encompassing healthcare personnel from various professions, investigating students, and researching healthcare staff from different settings and regions (especially more rural areas). This is crucial for a more comprehensive understanding of attitudes related to racism among the health workforce in Germany. Furthermore, investigating the attitudes of individuals in leadership positions in healthcare institutions is paramount, given their significant influence on institutional organization and the promotion of anti-racism interventions [15, 18]. Merz et al. suggest that beyond offering anti-racist interventions to healthcare staff, support staff (e.g., receptionists and nursing aides) should also be included to reduce implicit bias in non-clinical decision-making (room allocation, waiting times). They also highlight the importance of addressing racism carried out by fellow patients. Thus, expanding the investigation to include support staff and fellow patients also seems important.

## Appendix

### Interview Guide

#### Semantic Question

- S1:What do you understand by racism?

#### Episodic Questions

- E1: Do you observe situations where employees or patients experience racism in the hospital? Can you tell me about specific situations to illustrate this?
- E2: Have you ever thought about whether you yourself (in your work) think or act in a racist manner?
  - Possible follow-up questions:
    - When did you first think about this? What was the occasion?
    - Can you report on specific (current) situations where you felt that employees or patients were discriminated against by you in a racist manner?
    - How did you become aware that you may have acted in a racist manner?
  - Possible follow-up question:
    - What consequences did you draw from this?
- E3: Do you have the impression that your self-reflection on racism has changed over the course of your life? Can you give me examples?
  - Possible follow-up questions:
    - What do you attribute this change in your self-reflection to?
- E4: Are there people in your environment with whom you talk about your thoughts or feelings regarding racism?
- E5: Do you talk to colleagues about racism or racist discrimination at work?
- E6: Have patients ever approached you with this issue?

#### Semantic Question

- S2: What do you understand by privileges?

#### Episodic Question

- E7: How would you position yourself socially in terms of privileges?

#### Semantic Question

- S3: What do you understand by power?

#### Episodic Questions

- E8: To what extent do you feel you hold a position of power as a doctor in the hospital? Can you please tell me about one or more situations that illustrate this?
- E9: How strongly would you say your attitudes and actions affect your employees? Please tell me an example that makes this clear.
- E10: How significant is the impact of your attitudes and actions on your patients? Can you tell me about a situation that makes this clear?

#### Closing Questions

- Would you wish for anything regarding racism and the associated self-reflection?
- Is there anything else you would like to add that we have not yet discussed?

## Footnotes

1 The term “white” used to describe people is a socially constructed category with no biological basis. External characteristics are attributed to certain individuals and linked to assumed character traits and social norms, leading to power and privilege disparities. The asterisk (white*) is used to highlight this constructed nature. In line with Tißberger [16], this linguistic strategy, similar to the gender gap approach aimed at addressing heteronormativity and sexism, is applied here to address racism.

2 “General Data Protection Regulation” Law of the EU.

3 A = attendee; L = line.
